# Excellent Electrocatalytic Hydrogen Evolution Reaction Performances of Partially Graphitized Activated-Carbon Nanobundles Derived from Biomass Human Hair Wastes

**DOI:** 10.3390/nano12030531

**Published:** 2022-02-03

**Authors:** Sankar Sekar, Dae Hyun Sim, Sejoon Lee

**Affiliations:** 1 Department of Semiconductor Science, Dongguk University-Seoul, Seoul 04620, Korea; sanssekar@gmail.com (S.S.); gee04143@hanmail.net (D.H.S.); 2 Quantum-Functional Semiconductor Research Center, Dongguk University-Seoul, Seoul 04620, Korea

**Keywords:** biomass, activated carbon, nanobundles, electrocatalysts, hydrogen evolution reaction

## Abstract

Carbonaceous materials play a vital role as an appropriate catalyst for electrocatalytic hydrogen production. Aiming at realizing the highly efficient hydrogen evolution reaction (HER), the partially graphitized activated-carbon nanobundles were synthesized as a high-performance HER electrocatalyst by using biomass human hair ashes through the high-temperature KOH activation at two different temperatures of 600 and 700 °C. Due to the partial graphitization, the 700 °C KOH-activated partially graphitized activated-carbon nanobundles exhibited higher electrical conductivity as well as higher textural porosity than those of the amorphous activated-carbon nanobundles that had been prepared by the KOH activation at 600 °C. As a consequence, the 700 °C-activated partially graphitized activated-carbon nanobundles showed the extraordinarily high HER activity with the very low overpotential (≈16 mV at 10 mA/cm^2^ in 0.5 M H_2_SO_4_) and the small Tafel slope (≈51 mV/dec). These results suggest that the human hair-derived partially graphitized activated-carbon nanobundles can be effectively utilized as a high-performance HER electrocatalyst in future hydrogen-energy technology.

## 1. Introduction

Hydrogen (H_2_) has garnered tremendous attention because of its high energy efficiency, sustainability, recyclability, zero carbon emission, eco-friendliness, and alternativeness for fossil fuels [1,2,3,4]. For highly efficient and renewable hydrogen production, the hydrogen evolution reaction (HER) is of great importance [5,6,7,8,9]. In general, platinum (Pt)-based alloys are typically used as a HER electrocatalyst. However, both the low availability and the high cost of Pt have restricted its wide applications [10]. To realize future green and clean hydrogen energy technology, therefore, developing the low-cost and highly efficient HER electrocatalyst is essential. In recent years, various carbonaceous materials (e.g., graphene [11,12], activated carbon [13,14,15,16], graphene oxide [17], carbon nanotube [18,19,20,21], graphitic carbon [22], porous carbon [23,24], carbon fiber [25], etc.) have been studied in order to find an appropriate HER electrocatalyst that can replace the Pt alloys [26]. Among them, biomass activated carbon (AC) has become one of the adequate candidates as a HER electrocatalyst because of its high porosity, high conductivity, large surface area, high durability, high abundance, cost-effectiveness, and environmental friendliness [5,20,27]. For instance, Saravanan et al. [14] synthesized the AC nanosheets from peanut shells through the KOH activation, and they achieved an over potential of 80 mV at 10 mA/cm^2^ and a Tafel slope of 75 mV/dec. Prabu et al. [15] used the Ooty Varkey food wastes to derive the nanoporous AC sheets, possessing a good HER activity with a low overpotential of 380 mV at 10 mA/cm^2^ and a Tafel slope of 85 mV/dec. In addition, Liu et al. [24] prepared the N- and S-codoped porous carbon nanosheets by using human hair through the ZnCl_2_ activation at nitrogen atmosphere, and the nanosheets showed the outstanding HER characteristics with a low overpotential of 12 mV and a Tafel slope of 57.4 mV/dec at 10 mA/cm^2^. Among various biomass resources, because of its large carbon contents (>51% [28]), human hair can be utilized as an effective precursor for the derivation of carbon resources. Therefore, the reuse of the abundant biomass human hair wastes can be of superior benefit to fabricate the high-performance AC nanostructures. According to our best survey, however, the HER performance of human hair-derived activated carbon (HH-AC) has rarely been studied, except for a previous work by Liu’s group [24].

In light of these backgrounds, we investigated the facile and cost-effective synthesis of the partially graphitized mesoporous AC nanobundles by using biomass HH via the high-temperature KOH activation and characterized their structural, morphological, textural, and electrocatalytic properties. Herein, we report on the state-of-the-art HER activities of the HH-AC layered-nanobundles (i.e., extremely low overpotential of ~16 mV at 10 mA/cm^2^ in 0.5 M H_2_SO_4_ and a very small Tafel slope of ~51 mV/dec).

## 2. Experimental Section

### 2.1. Synthesis of HH-AC Nanobundles

**Figure 1:**
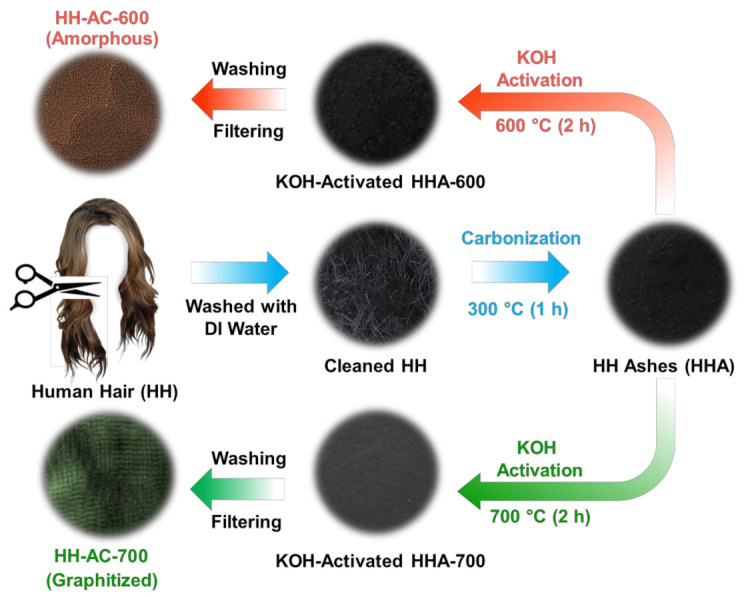
Schematic illustration of the synthesis process for the HH-AC-600 nanobundles and the HH-AC-700 layered nanobundles.

Figure 1 schematically illustrates the experimental procedure for the synthesis of biomass HH-AC. The biomass carbonaceous resource of HH (100 g) was collected from the lead authors participated in this work. Firstly, a bundle of HH was cleaned in deionized (DI) water and dried in air atmosphere during 12 h. To obtain the carbonaceous precursors, thereafter, DI-cleaned HH was carbonized at 300 °C for 1 h in air. Subsequently, 3 g of HH-carbonized ash (HHA) was mixed with 12 g of KOH (Sigma Aldrich, Seoul, Korea) in a ceramic mortar and annealed at two different temperatures of 600 and 700 °C for 2 h in air. After the KOH activation, the mixture of KOH-HHA was soaked in 100 mL DI water for 10 h to remove the potassium-based precipitates. 

Finally, the powder types of amorphous HH-AC (KOH-activated at 600 °C) and graphitized HH-AC (KOH-activated at 700 °C) were collected, filtered, washed, and dried at 150 °C for 8 h. For convenience, we denoted the former and the latter as ‘HH-AC-600’ and ‘HH-AC-700’, respectively.

### 2.2. Material Characterizations

The morphological and compositional properties of the HH-AC-600 and HH-AC-700 samples were assessed through the field-emission scanning-electron microscopy (FE-SEM) and in situ energy dispersive X-ray (EDX) spectroscopy measurements, respectively, by using an Inspect-F50 system (FEI, Mahwah, NJ, USA). Additionally, the microstructural properties of the samples were further investigated through the transmission electron microscopy (TEM) and in situ selective-area electron diffraction (SAED) measurements by using a JEM 2100F system (JEOL, Tokyo, Japan). The crystallographic and the vibrational characteristics were analyzed by the X-ray diffraction (XRD) and Raman scattering spectroscopy measurements, respectively, by using a D8-Advance system (Bruker, Madison, WI, USA) and a LabRAM HR800 system (Jobin Yvon, Longjumeau, France). The textural properties of the samples were examined via the Brunauer–Emmett-Teller (BET) and Barrett–Joyner–Halenda (BJH) analysis methods by using a BELSORP-mini II equipment (MicrotracBEL, Osaka, Japan). To gain a reliability of the materials characterization, for all measurements, we carried out at least three runs by using multiple samples that were fabricated by the identical synthesis processes.

### 2.3. Electrocatalytic Measurements

To characterize the electrocatalytic performances of HH-AC-600 and HH-AC-700, as a primary task, the working electrodes were fabricated by using the HH-AC-600 and HH-AC-700 samples. First, activated carbon (e.g., either HH-AC-600 or HH-AC-700) was added into the N-methyl-2-pyrrolidinone solution. Then, the mixture slurries were coated on the stainless steel substrates (1 cm^2^) and dried at 150 °C for 8 h. In addition, the saturated calomel electrodes (SCE) and the platinum meshes were used as a reference electrode and a counter electrode, respectively. The HER activity of the HH-AC-600 and HH-AC-700 electrocatalysts were investigated by linear sweep voltammetry (LSV) and cyclic voltammetry (CV) via the three-electrode method using a VersaSTAT-3 electrochemical workstation (Ametek Scientific Instruments, Berwyn, PA, USA). The CV measurements were carried out under various potential scan rates from 10 to 100 mV/s within a potential window of 0–0.8 V. The LSV measurements were performed in a 0.5 M H_2_SO_4_ electrolyte solution under the constant potential scan rate of 5 mV/s. The electrochemical impedance spectroscopy (EIS) measurements were conducted at a frequency range of 1 Hz–10 kHz. In 0.5 M H_2_SO_4_, the overpotential (η) and the Tafel slope (S*_T_*) were determined by using the equations below:

(1) $$E_{RHE}\;=\;E_{SCE}\;+\;0.26\;V$$

(2) $$ɳ\;=\;S_{T}\log\;\left(J\right)\;+\;c$$

where E*_RHE_* and E*_SCE_* are the standard potential values of the reversible hydrogen electrode and the saturated calomel electrode, respectively. *c* is the fitting parameter.

## 3. Results and Discussion

**Figure 2:**
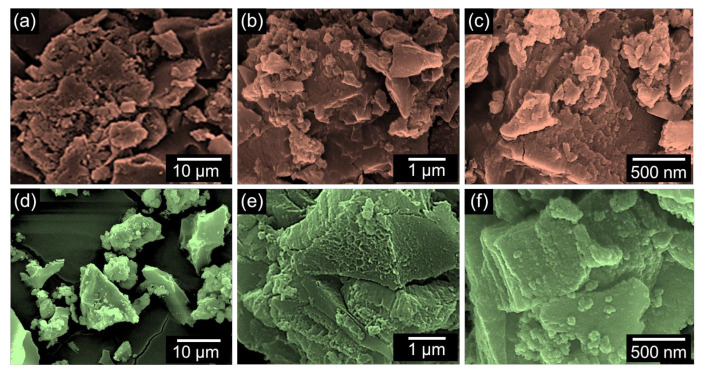
FE-SEM images of (**a**–**c**) HH-AC-600 and (**d**–**f**) HH-AC-700.

**Figure 3:**
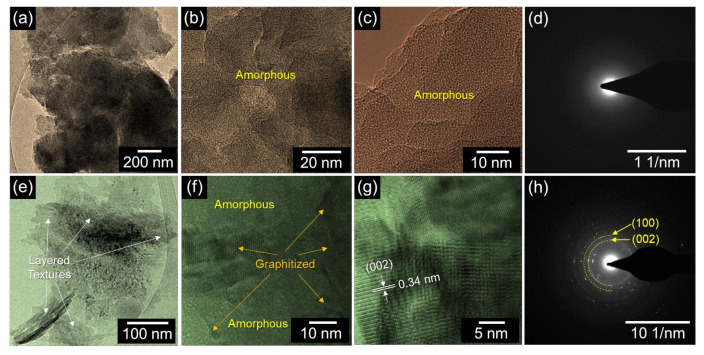
(**a**) Bright-field TEM image, (**b**,**c**) high-resolution TEM images, and (**d**) SAED pattern of HH-AC-600; (**e**) bright-field TEM image, (**f**,**g**) high-resolution TEM images, and (**h**) SAED patterns of HH-AC-700.

Figure 2 shows the FE-SEM images of the HH-AC-600 and HH-AC-700 samples. The HH-AC-600 sample displays the nanobundles-aggregated morphology (Figure 2a–c). Similarly, the HH-AC-700 sample also exhibits the aggregated nanobundles-like morphology (Figure 2d–f). In the case of HH-AC-700, however, the layered texture became clearer than HH-AC-600. From the EDX analysis, both samples were confirmed to contain only their main species of C (see Figure S1, Supplementary Materials). The microstructural insights of the HH-AC-600 and the HH-AC-700 samples were further monitored by TEM. As shown in Figure 3a, HH-AC-600 reveals the aggregated nanobundles-like structure. From the high-resolution TEM images (Figure 3b,c) and the SAED pattern (Figure 3d), one can specify that the HH-AC-600 nanobundles are amorphous. In the case of HH-AC-700, the aggregated nanobundles are also observable from its bright-field TEM image (Figure 3e). Differently from HH-AC-600, however, the stacked/layered nanobundles are much more clearly visible. The average thickness of the layered nanobundle stack is ~20 nm. Here, it should be noticed that the layered HH-AC-700 nanobundles display the partially graphitized crystallites, although some parts still remain amorphous (Figure 3f). The lattice spacing of the crystallite is 0.34 nm (Figure 3g); and the lattice planes of the crystallites are (002) and (100), as depicted in the SAED pattern (Figure 3h).

**Figure 4:**
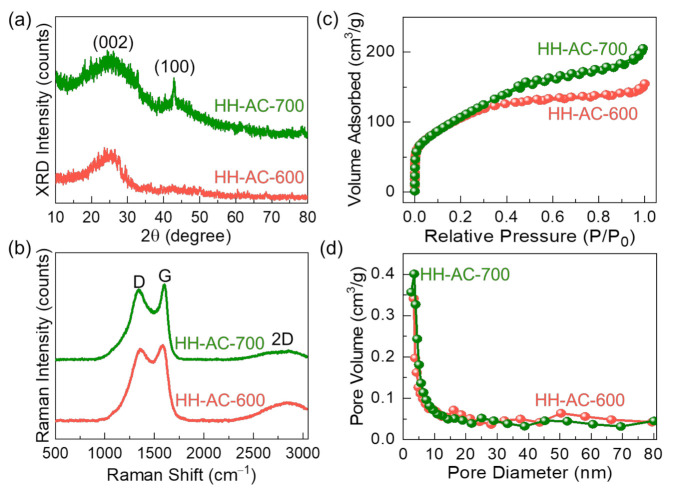
(**a**) XRD patterns, (**b**) Raman spectra, (**c**) N_2_ adsorption–desorption isotherm characteristics, and (**d**) pore size distribution characteristics of HH-AC-600 and HH-AC-700.

Next, the crystallographic properties of HH-AC-600 and HH-AC-700 were examined by XRD. As shown in Figure 4a, the samples reveal a wide diffraction pattern at the Bragg angle at 24.8° with a small Bragg pattern at 42.6°. The former and the latter correspond to the (002) and the (100) lattice planes of the disordered carbon structure [29,30,31], respectively. For the HH-AC-700 nanobundles, the (100) intensity is much stronger than that of the HH-AC-600 nanobundles. This infers that the HH-AC-700 nanobundles contain a high density of pores exclusive to the solid-state graphitic carbon [32,33]. These results indicate that the HH-AC-700 nanobundles have a good quality for the electrocatalysts electrode material (e.g., high surface area, high porosity, high conductivity, etc.).

The graphitic nature was further elucidated by the Raman scattering spectroscopy measurements. Both HH-AC-600 and HH-AC-700 samples display three predominant Raman bands at 2848 cm^−1^ (2D band), 1601 cm^−1^ (G band), and 1345 cm^−1^ (D band) (Figure 4b). The 2D band is indicative of the distinctive signature from the activated carbon; and the G and the D bands are attributed to the E_2g_ vibration mode of the sp^2^ hybridized carbon and the disorder vibration mode of graphitic carbon, respectively [34,35,36]. According to previous reports [29], the I_D_/I_G_ ratio represents the degree of graphitization for the layered carbonaceous materials. The I_D_/I_G_ ratio was determined to be 0.96 and 0.93 for HH-AC-600 and HH-AC-700, respectively. Therefore, one can conjecture that, in comparison with the amorphous HH-AC-600 nanobundles, the partially graphitized HH-AC-700 nanobundles have a higher degree of graphitization and a lower degree of the disordered lattice configuration.

The graphitization of AC would strongly affect the porosity of the entire material system because the local recrystallization at the high temperature gives rise to the increase in structural faults and voids at both the crystallite interfaces and the grain boundaries inside the partially crystallized solid-state material system (i.e., polycrystalline-amorphous mixture) [31,37]. To verify such a hypothesis, the textural properties were assessed through the BET and the BJH methods. Figure 4c shows the N_2_ adsorption–desorption isotherm characteristic curves of HH-AC-600 and HH-AC-700. Both samples clearly reveal the Type-IV isotherm feature with a Type-H4 hysteresis loop, which can be classified from IUPAC [34], indicating the mesoporous characteristics of the prepared materials [29,38,39]. From the BET analysis, the specific surface areas (S_ss_) of HH-AC-600 and HH-AC-700 were determined to be 684 and 936 m^2^/g, respectively. Through the BJH analysis (Figure 4d), additionally, the pore surface areas (S_ps_) of HH-AC-600 and HH-AC-700 were calculated to be 204 and 231 m^2^/g, respectively. Compared to the HH-AC-600 nanobundles (average pore size (d_ap_) ≈ 4.52 nm, total pore volume (V_tp_) ≈ 0.195 cm^3^/g), accordingly, the d_ap_ and V_tp_ values of the HH-AC-700 nanobundles were decreased down to 3.47 nm and increased up to 0.237 cm^3^/g, respectively. These mean that the partially graphitized HH-AC-700 nanobundles have a higher porosity than that of the amorphous HH-AC-600 nanobundles. Therefore, one can surmise that the microstructural modification (i.e., partial graphitization) of the HH-AC-700 nanobundles via the higher temperature KOH activation could lead to the increase in both the textural porosity and the electrical conductivity.

**Figure 5:**
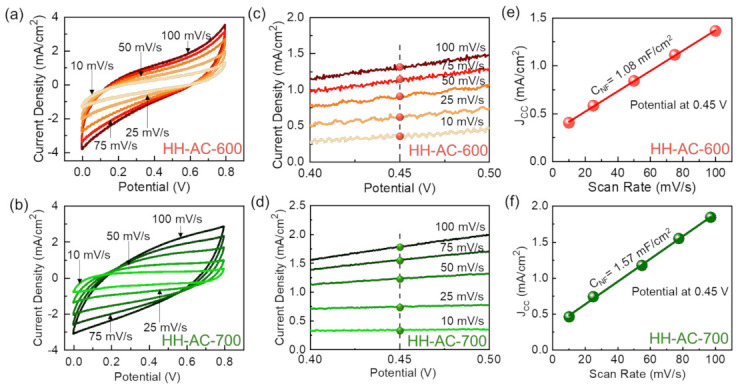
CV curves of the (**a**) HH-AC-600 and the (**b**) HH-AC-700 electrodes. CV curves at the Non-Faradaic region near 0.45 V for the (**c**) HH-AC-600 and the (**d**) HH-AC-700 electrodes. J*_CC_* as a function of the scan rate for the (**e**) HH-AC-600 and the (**f**) HH-AC-700 electrodes.

In electrocatalysts, both the enhanced porosity and the increased conductivity play crucial roles for improving the electrochemical reaction. To characterize the electrocatalytic activity of HH-AC-600 and HH-AC-700, thus, the CV properties were measured by using a three-electrode system. As can be seen from Figure 5a,b, both samples exhibit the large CV hysteresis loops at various scan rates of 10–100 mV/s. Such a clear appearance of the typical rectangular CV loops indisputably depicts the effective electrocatalytic behavior occurring inside the electrocatalyst [36,40,41]. Compared to HH-AC-600, however, HH-AC-700 show the higher integrated CV areas and the improved current–voltage responses. This corroborates that the partially graphitized HH-AC-700 nanobundles hold higher porosity and superior electrical conductivity than those of the amorphous HH-AC-600 nanobundles.

The electrocatalytic HER performance is closely related to the electrochemically active surface area (*ECSA*) of the active electrode material, at which the acidic solutions could react with AC, resulting in the hydrogen production via the chemical reaction of ‘AC + 2H^+^ + 2e^−^ → H_2_′ [4,42]. From the non-Faradic CV region, one can easily estimate the *ECSA* value by using the following equations [5,27,43]:

(3) $$ECSA\;=\;C_{NF}/C_{E}$$

(4) $$J_{CC}\;=\;C_{NF}\;\times \;v/A$$

where C*_NF_*, C*_E_*, J*_CC_*, *v*, and A are the non-Faradic capacitance, the electrolyte capacitance (0.035 mF/cm^2^ for 1 M H_2_SO_4_) [44,45,46], the charging current density, the scan rate, and the electrode area, respectively. Figure 5c–f display the non-Faradic CV curves at 0.4–0.5 V and their corresponding J*_CC_* values at 0.45 V as a function of the scan rate. By fitting those experimental data to the above equations, the *ECSA* values were calculated to be 61 and 89 cm^2^ for HH-AC-600 and HH-AC-700, respectively, and these values are comparable to and even greater than literature values of other carbonaceous HER electrocatalysts [46,47]. This signifies that the active HH-AC-700 electrode material (i.e., partially graphitized mesoporous HH-AC nanobundles) has the improved ion-storage capacity as well as the enhanced electrocatalytic activity compared to the HH-AC-600 electrode.

**Figure 6:**
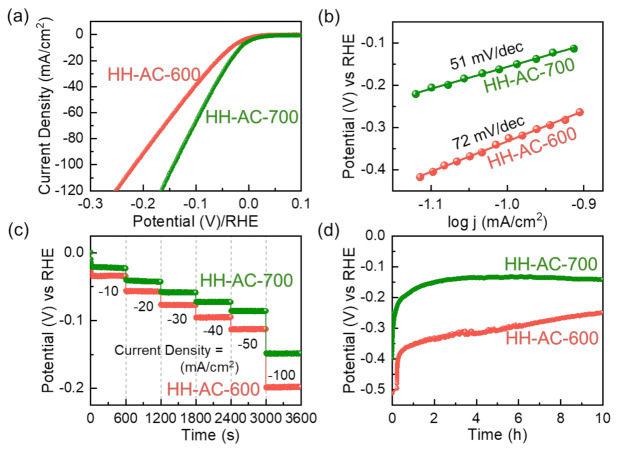
(**a**) LSV polarization curves, (**b**) Tafel curves, (**c**) multi-chronopotentiometric profiles at various current densities (from −10 to −100 mA/cm^2^), and (**d**) time-dependent HER durability for the HH-AC-600 and the HH-AC-700 electrodes.

Based upon all the above results, one may expect the partially graphitized mesoporous HH-AC-700 nanobundles to act as an effective HER electrocatalyst because the enhanced electrocatalytic activity is one of the most necessary conditions for the high-performance HER electrode [20,23]. To attest this, the electrocatalytic HER performances of the HH-AC-600 and HH-AC-700 electrodes were examined by measuring the LSV characteristics at 5 mV/s in 0.5 M H_2_SO_4_. From the obtained polarization LSV curves (Figure 6a), the HH-AC-600 and HH-AC-700 electrodes were confirmed to have the lower η values of 34 and 16 mV at 10 mA/cm^2^, respectively. 

Furthermore, the samples showed the enhanced electrocatalytic reaction kinetics. Namely, the S*_T_* values (Figure 6b) of the HH-AC-600 and HH-AC-700 electrodes were calculated to be 72 and 51 mV/dec, respectively. Compared to the HH-AC-600 electrode, the HH-AC-700 electrode has a superior HER electrocatalytic performance (i.e., lower overpotential: 16 mV at 10 mA/cm^2^ and smaller Tafel slope: 51 mV/dec) because of its high surface area, large porosity, high electrical conductivity, and electrochemically active surface area. In additional, such an excellent HER activity of HH-AC-700 is comparable to the state-of-the art carbonaceous HER electrocatalyst (i.e., HH-derived N- and S-codoped porous AC [24]) and is far superior to any others ever reported (see Table 1). Figure 6c displays the chronopotentiometric characteristics of the HH-AC-600 and HH-AC-700 electrodes. The multiple chronopotentiometric characteristics reveal that the HH-AC-700 electrode has a smaller overpotential at each current density than that of the HH-AC-600 electrode. Additionally, the HH-AC-700 electrode exhibits the stable and long-term HER durability, compared to the HH-AC-600 electrode (Figure 6d). After the durability test, the overpotential was further reduced because the electrical current might galvanize the material. Furthermore, the LSV curves before and after the durability test clearly demonstrated their outstanding electrochemical stability for the HER performance in the H_2_SO_4_ solution (Figure S2).

**Figure 7:**
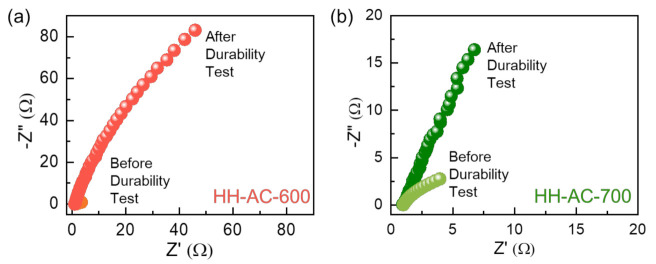
Nyquist plots of the (**a**) HH-AC-600 and the (**b**) HH-AC-700 electrodes before and after the durability test.

To elucidate the kinetics of the excellent HER activity, the EIS measurements were conducted before and after the durability test. As shown in Figure 7a,b, regardless of the durability test, both electrodes reveal only a linear EIS feature, arising from the effective electrolyte diffusion into the electrode [48,49]. Here, one needs to focus on the absence of semicircles at the high frequency region. Since the semicircle is associated with the charge transfer resistance [25,50], no semicircle in the EIS spectra infers that the HER catalyst has a high electronic conductivity and a high ionic diffusivity [5,27]. The series resistance (R_s_ ≈ 1 Ω) value is smaller for the HH-AC-700 nanobundles than that for the HH-AC-600 nanobundles (R_s_ ≈ 2.4 Ω). Consequently, the partial graphitization of the HH-AC-700 nanobundles gives rise to the increases in both the textural porosity and the electrical conductivity and eventually leads to the superior HER activity via the enhanced ionic diffusion and the effective electronic charge transport.

## 4. Conclusions

The HH-AC nanobundles were effectively synthesized by the KOH activation of the biomass HH carbonaceous resources. The 600 °C-activated HH-AC nanobundles showed the amorphous phase, whereas the 700 °C-activated HH-AC nanobundles displayed the partially graphitized crystallite textures. Since the partial graphitization of AC increases both the textural porosity (average pore size ≈ 3.47 nm, total pore volume ≈ 0.237 cm^3^/g) and the electrical conductivity (series resistance ≈ 1 Ω), the 700 °C-activated HH-AC sample showed the superior HER activity with an extremely low overpotential (≈16 mV at 10 mA/cm^2^ in 0.5 M H_2_SO_4_) and a very small Tafel slope (≈51 mV/dec). The results suggest that the partially graphitized nanobundles can be utilized as an excellent HER electrocatalyst for future green hydrogen-energy technology.

## Figures and Tables

**Table 1:** Comparison of the HER performances for the proposed HH-AC-600 and HH-AC-700 with other carbonaceous electrocatalysts reported in previous works.

Catalyst	Overpotential η (mV)	Tafel Slope S*_T_* (mV/dec)	Electrolytes	Reference
HH-AC-700 layered nanobundles	16	51	0.5 M H_2_SO_4_	This Work
HH-AC-600 nanobundles	34	72	0.5 M H_2_SO_4_	This Work
N- and S-codoped graphene	276	81	0.5 M H_2_SO_4_	[11]
N- and P-codoped graphene	106	67.3	0.5 M H_2_SO_4_	[12]
Defective AC	334	66	0.5 M H_2_SO_4_	[13]
N-doped AC	80	75	0.5 M H_2_SO_4_	[14]
Nanoporous AC	380	85	0.5 M H_2_SO_4_	[15]
N- and S-codoped AC	450	163	1 M KOH	[16]
N- and S-codoped CNT *	450	133	1 M KOH	[18]
Ni-NiO-CNT composite	276	94	1 M H_2_SO_4_	[19]
Activated CNT *	225	71	0.5 M H_2_SO_4_	[20]
N-doped porous carbon	179	98	1 M KOH	[23]
N- and S-codoped porous carbon	12	57.4	0.5 M H_2_SO_4_	[24]
N-doped carbon fiber	150	89	0.5 M H_2_SO_4_	[25]

* Note. CNT: carbon nanotube.

## Data Availability

The data presented in this study are available on request from the corresponding author.

## Supplementary Materials

The following supporting information can be downloaded at: https://www.mdpi.com/article/10.3390/nano12030531/s1, Figure S1: EDX spectra of the (a) HH-AC-600 and (b) HH-AC-700 samples. The small amounts of Pt arose from the ultrathin Pt conducting layer, which had been coated on the samples for better visualization of the FE-SEM images, Figure S2: LSV curves of the (a) HH-AC-600 and (b) HH-AC-700 electrodes for HER before and after the durability test.
