# Supplementary material for: Excellent Electrocatalytic Hydrogen Evolution Reaction Performances of Partially Graphitized Activated-Carbon Nanobundles Derived from Biomass Human Hair Wastes
Source: Nanomaterials (Basel). 2022 Feb 3;12(3):531. doi: 10.3390/nano12030531 (PMC8838363; doi:10.3390/nano12030531)
Supplement: Supplementary file 1 [file nanomaterials-12-00531-s001.zip › nanomaterials-1567952-supplementary.pdf]

nanomaterials

Supplementary Materials

Excellent Electrocatalytic Hydrogen Evolution Reaction Performances of Partially-Graphitized Activated-Carbon Nanobundles-Derived from Biomass Human Hair Wastes

Sankar Sekar [1,2], Dae Hyun Sim [1,2] and Sejoon Lee [1,2,*]

[1] Department of Semiconductor Science, Dongguk University-Seoul, Seoul 04620, Korea; sanssekar@gmail.com (S.S.); gee04143@hanmail.net (D.H.S.)
[2] Quantum-functional Semiconductor Research Center, Dongguk University-Seoul, Seoul 04620, Korea
* Correspondence: sejoon@dongguk.edu

1. Elemental Characteristics of HH-AC

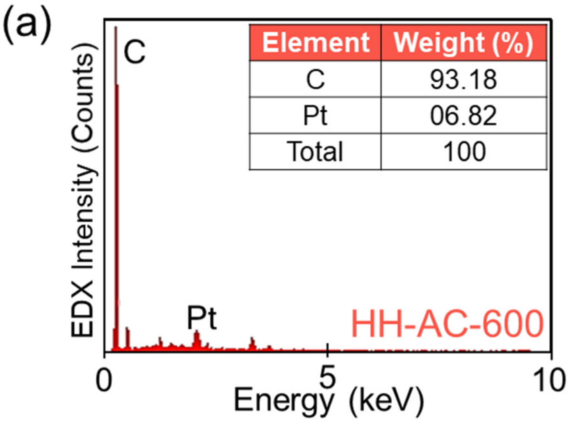

Figure S1. EDX spectra of the (**a**) HH-AC-600 and (**b**) HH-AC-700 samples. The small amounts of Pt arose from the ultrathin Pt conducting layer, which had been coated on the samples for better visualization of the FE-SEM images.

2. Electrochemical Properties of HH-AC before and after Durability Test

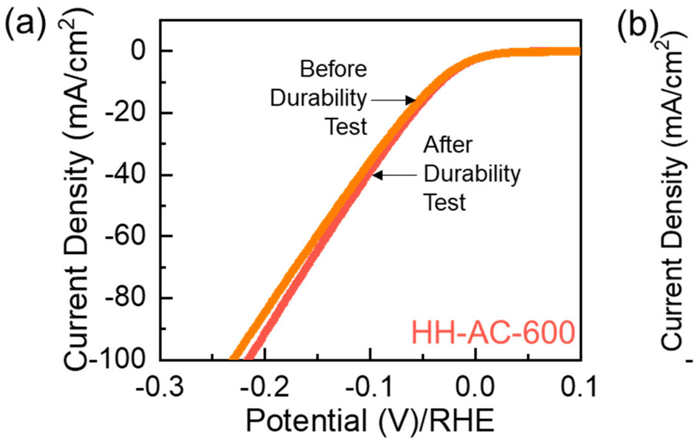

Figure S2. LSV curves of the (**a**) HH-AC-600 and (**b**) HH-AC-700 electrodes for HER before and after the durability test.
